# Association between ТР53, MDM2 and NQO1 gene polymorphisms and viral load among women with human papillomavirus

**DOI:** 10.18699/VJGB-22-09

**Published:** 2022-02

**Authors:** A.H. AlBosale, E.V. Mashkina

**Affiliations:** Al-Dour Technical Institute, Northern Technical University, Department of Medical Laboratory Techniques, Mosul, Iraq; Southern Federal University, Rostov-on-Don, Russia; Southern Federal University, Rostov-on-Don, Russia

**Keywords:** polymorphism, human papillomavirus, viral load, TP53, MDM2, NQO1, полиморфизм, вирус папилломы человека, вирусная нагрузка, TP53, MDM2, NQO1

## Abstract

The risk of cervical cancer is caused by persistent human papillomavirus (HPV) infection. Cervical cancer is the most frequent cancer among women. Our purpose was to investigate the association between TP53 215C>G (Pro72Arg), MDM2 -410T>G, and NQO1 609C>T gene polymorphisms with a high HPV load and the inf luence of gene-gene interactions on prolonged HPV infection. Eighty-nine women with a high HPV viral load and 114 healthy women were involved in a case–control study. Genotyping for TP53 215C>G (Pro72Arg) and MDM2 -410T>G SNPs was carried out by allele-specif ic PCR and genotyping for NQO1 609C>T was performed by a TaqMan assay. Quantitative analysis of HPV DNA was performed by AmpliSens® HPV HCR screen-titer-FRT test system. Gene-gene interactions were analyzed using the multifactor dimensionality reduction (MDR) method. The study of separate SNPs of MDM2 -410T>G and NQO1 609C>T genes did not reveal any statistically signif icant difference in genotype and allele frequencies among women within the two groups. The frequency of the 215G (72Arg) allele and 215GG (72Arg/ Arg) genotype of the TP53 gene was signif icantly higher in the case group than in the control group (OR = 1.74, 95 % CI = 1.10–2.73; p = 0.02 and OR = 1.97, 95 % CI = 1.13–3.46; p = 0.04, respectively). MDR analysis showed the signif icance of intergenic interactions of the three studied loci TP53 (rs1042522) – MDM2 (rs2279744) – NQO1 (rs1800566) for the formation of a high HPV load (OR = 3.05, 95 % CI = 1.73–5.46; p = 0.0001).

## Abstract (ru)

Риск рака шейки матки вызван персистирующей инфекцией вируса папилломы человека (ВПЧ). Наша цель – исследовать связь между полиморфизмами генов TP53 215C>G (Pro72Arg), MDM2 -410T>G и NQO1 609C>T с риском формирования высокой вирусной нагрузки при ВПЧ-инфекции. Восемьдесят девять женщин с высокой вирусной нагрузкой ВПЧ и 114 здоровых женщин были вовлечены в исследование случай–контроль. Генотипирование для SNP TP53 Pro72Arg и MDM2 -410T>G проводили методом аллель-специфичной ПЦР, а для NQO1 609C>T – путем анализа ПЦР в реальном времени с использованием TaqMan зондов. Количественный анализ ДНК ВПЧ выполняли с использованием тест-системы «АмплиСенс ВПЧ ВКР скрин-титр-FL». Анализ межгенных взаимодействий осуществляли с помощью алгоритма многофакторного снижения размерности (MDR). Исследование отдельных SNP генов, MDM2 -410T>G и NQO1 609C>T, не выявило статистически значимой разницы в частотах генотипов и аллелей среди женщин в двух группах. Частота аллеля 72Arg и генотипа 72Arg/Arg гена TP53 в группе ВПЧ-инфицированных женщин была значительно выше, чем в контрольной группе (OR = 1.74, 95 % CI = 1.10–2.73; p = 0.02 и OR = 1.97, 95 % CI = 1.13–3.46; p = 0.04 соответственно). MDR-анализ показал значимость межгенных взаимодействий исследуемых локусов TP53 (rs1042522) – MDM2 (rs2279744) – NQO1 (rs1800566) для формирования высокой нагрузки ВПЧ (OR = 3.05, 95 % CI = 1.73–5.46; p = 0. 0001).

## Introduction

Human papillomavirus (HPV) is implicated in the development of cervical cancer. A key critical step in papillomavirusrelated carcinogenesis is a persistent viral infection (Vonsky et al., 2019). There is heterogeneity in the development of human papillomavirus infection due to genetic variations, ethnicity, viral types involved in infection, viral load, and oncogenic expression, as well as environmental, and hormonal, physiological, and nutritional factors (Roura et al., 2016; Tasic et al., 2018). After HPV-infection, especially with high-risk HPV types (16, 18, 31, 33, 35, 39, 45, 51, 52, 56, 58, and 59), HPV oncoproteins induce mutations in oncogenes, epigenetic modifications, and chromosomal rearrangements (Mittal, Banks, 2017). A disequilibrium in the relationship between virus and host results in a decrease in the effectiveness of the immune system, the imbalance between cellular and humoral immune processes, as well as alteration in pro- and antiinflammatory cytokine levels, which increases the replicative ability of the virus (Fernandes et al., 2015; Bordignon et al., 2017). In addition, modifications that alter the stability of cell cycle proteins such as retinoblastoma protein (pRb), tumor suppressor p53, result in uncontrolled cell cycle progression and induce oncogenic transformation of cells (Sen et al., 2017; Balasubramaniam et al., 2019).

The TP53 tumor suppressor gene plays a crucial role in regulating DNA repair, apoptosis, and cell cycle control. It has been observed that most human tumors contain mutated p53, with about 50 % of those mutations causing a reduction in DNA repair ability, irregular cell growth, and, eventually, progression to malignancy (Aubrey et al., 2016). Polymorphisms in the TP53 gene change p53 protein conformation, which leads to p53 degradation through a process mediated by ubiquitin (Rampias et al., 2014). The most widely studied of the non-synonymous SNP TP53 Pro72Arg (rs1042522) replaces proline (Pro) with arginine (Arg) in the p53 protein due to a substituted C to G base in the TP53 gene. Both variants have the same binding affinity for DNA while their ability to bind components of the transcription factor is different. So, the two variants of the p53 protein are not functionally equivalent (Thomas et al., 1999). The p53 is ubiquitinated in the proteasome, which is regulated by MDM2 via a ubiquitin-dependent degradation pathway and NAD(P)H quinone oxidoreductase 1 via a ubiquitin-independent degradation pathway (Tsvetkov et al., 2010; Karni-Schmidt et al., 2016). As a result, the level of p53 is affected by MDM2 and NQO1 activity.

Oncoprotein MDM2 is a negative regulator of the p53 tumor protein (Saadatzadeh et al., 2017). A functional SNP in the MDM2 gene promoter (-410T>G rs2279744) regulates MDM2 protein expression. When T is replaced with G, this increases the affinity of the transcriptional activator Sp1, resulting in higher MDM2 expression and subsequent suppression of the p53 pathway (Bond et al., 2004).

The NQO1 enzyme can catalyze the reduction of various quinones to hydroquinones by a two-electron reduction mechanism (NADH or NADPH) as a reducing cofactor. This two-electron reduction prevents the formation of free radicals (semiquinones) that protect the cells from oxidative stress (Atia, Abdullah, 2020). The SNP of NQO1 at nucleotide position 609C>T in exon 6 (rs1800566) with the proline to serine amino acid substitution at codon 187 induces a change of enzyme activity. The homozygotes (TT ) genotype gives rise to an inactive enzyme NQO1, heterozygotes (CT ) have the enzyme displays mild activity, while the wild homozygotes (CC) have the highest activity of the NQO1 (Ross, Siegel, 2004). Wild type NQO1 partially inhibits HPV E6-mediated p53 degradation, although this does not occur with the mutant type NQO1 (Niwa et al., 2005).

Thus, the efficiency of the cell cycle repair and control system depends not only on the p53 protein. Also, the levels of MDM2 and NQO1 proteins in the cell can affect the stability of the p53 protein and the activity of its degradation processes. However, human papillomavirus, as an exogenous factor, can be an additional cause affecting the work of the repair system. Most of the studies on the association of SNPs of genes with HPV infection and cervical cancer are devoted to the analysis of individual nucleotide substitutions. There is practically no data in the literature on the combined effect of polymorphic variants of these three genes in the presence of HPV load.

Our work aims to analyze the distribution of the polymorphisms of the TP53 gene (rs1042522), MDM2 gene (rs2279744), and NQO1 gene (rs1800566) in patients with HPV load versus HPV-negative women.

## Materials and methods

Two hundred and three samples of epithelial cells scraped from the urogenital tract of women were used for molecular genetic studies. The study equipment has been provided by the clinical diagnostic laboratory, Nauka (Rostov-on-Don, Russia). The women were divided into two groups: women with a high HPV load (above 3 log of HPV genomes per 100 thousand human cells) (n = 89), and HPV-negative women (n = 114). All the women included in the study were over thirty years old. Criteria for women being included in the control group: a normal result of colposcopy, HPV-negative PCR-test. The comparative group of cases included women with symptoms such as vaginal discharge, bleeding menstrual abnormalities, and HPV-positive PCR-test with an HPV load of more than 3 log of HPV genomes per 105 human cells. The ethnic composition of the women involved in the study groups was as follows: Russians accounted for 86 %, Armenians accounted for 9 %, and other nationalities of the Caucasian race – 5 %.

All women have given formal written consent to take part in the study. The study was approved by the Bioethics Committee of the Academy of Biology and Biotechnology of the Southern Federal University (Protocol No. 2 of March 29, 2016). All the tests for clinical experimentation were carried out in line with the standards and ethical guidelines of the World Medical Association (Helsinki Declaration).

The total DNA was isolated from scraping epithelial cells of the cervical canal of women according to the DNA-sorb-AM (NextBio, Russia) reagent kit protocol. The quantification of DNA for high-risk HPV types (16, 18, 31, 33, 35, 39, 45, 51, 52, 56, 58, and 59) in biological material was analyzed according to the AmpliSens-HPV HCR screen-titre-FRT PCR kit (Interlabservice, Russia) protocol. According to the kit manufacturer’s instructions and clinical reports, the viral load is interpreted as follows: log ≤ 3 per 105 human cells – low clinical significance, 3–5 log per 105 human cells – clinically significance, risk of dysplasia; and > 5 log per 105 human cells – clinically significance, strongly probable dysplasia.

Genotyping was performed for the SNP of TP53 215C>G (Pro72Arg) (rs1042522), MDM2 -410T>G (rs2279744) genes by allele-specific PCR and the SNP-express reagent (Lytech, Russia) according to the kit protocol. NQO1 609C>T (rs1800566) was genotyped by a TaqMan genotyping assay. The amplification was carried out in a 25-ml reaction containing 2 μl 25 mM MgCl2, 1 μl 10 mmol/L of the forward primer (5′-CAG AGT GGC ATT CTG CAT TTC T-3′) and reverse (5′-CTG GAG TGT GCC CAA TGC TA-3′) primers and 0.5 μl mmol/L NQO1 wild-type (5′-6FAM-CTT AGA ACC TCA ACT GA-MGBNFQ-3′) and mutant (5′-VIC-CTT AGA ATC TCA ACT GAC A-MGBNFQ-3′) probes, 0.5 μl Taqpolymerase (5 U/μl), 2.5 μl 2.5 мМ of dNTP, 2 μl 10 × PCR buffer B, 12 μl ddH2O (Syntol, Russia) and 3 μl DNA. Cycling conditions were as follows: initial denaturation at 95 °C for 10 min, followed by 40 cycles consisting of denaturation at 95 °C for 15 sec, then annealing at 54 °C for 60 sec. The PCR products for NQO1 609C>T (rs1800566) were analyzed in real-time using RotorGene thermocycler. PCR products for the TP53 Pro72Arg and MDM2 -410T>G genes were analyzed by 3 % agarose gel horizontal electrophoresis and visualized under the ultraviolet (UV) transilluminator GelDoc (Bio-Rad, USA).

To calculate the statistical data, the χ2 test was used to compare the allele and genotype frequencies of the TP53 215C>G (Pro72Arg) (rs1042522), MDM2 -410T>G (rs2279744), and NQO1 609C>T (rs1800566) genes in the case group and control group. The Hardy–Weinberg equilibrium test was performed to determine the goodness-of-fit of the χ2 test with one degree of freedom by comparing the observed genotype frequencies with the expected genotype frequencies. The SNP genetic association was assessed by the χ2 test, odds ratio (OR), and its confidence interval (CI). A p-value <0.05 was considered statistically significant. Statistical analyses were performed using GraphPad InStat 3.05 software.

The analysis of intergenic interactions was performed using the MDR software (http://www.multifactordimensionalityre duction.org/) and by using the exhaustive search algorithm. All potential combinations of genotypes were evaluated with respect to the risk of developing an HPV infection. Multilocus genotypes are summed up in the MDR program into groups of increased and reduced disease risk, which reduces the dimension of the number of calculated parameters. Using multiple cross-recalculations of the input primary data, the optimal model is selected for intergenic interaction, with the highest accuracy and, accordingly, with the least error, to predict the presence or absence of predisposition to the studied pathology.

## Results

In 89 HPV-positive women, the average age was 40.1 ± 7.3 years and 41.1 ± 7.6 years in 114 HPV-negative women. Among 89 HPV-infected women the minimum, middle, and maximum HPV DNA load were 3.2, 5.1, and 8.6 log of HPV genomes per 100 thousand human cells, respectively

Frequency distributions for the three investigated TP53, MDM2, and NQO1 gene polymorphisms are given in Table 1. The polymorphic variants of MDM2 -410T>G and NQO1 609C>T were not associated with a high HPV load. At the same time females with a high HPV load had a significantly higher frequency of TP53 215G (72Arg) allele (OR = 1.74, 95 % CI = 1.10–2.73; p = 0.02) and 215GG (72ArgArg) genotype (OR = 1.97, 95 % CI = 1.13–3.46; p = 0.04) than healthy controls. The existence of multiple allelic variations in genes that encode for protein molecules can lead to several related changes in the genome and proteome function. Therefore, an analysis of intergenic interactions of allelic variants was conducted.

**Table 1.**
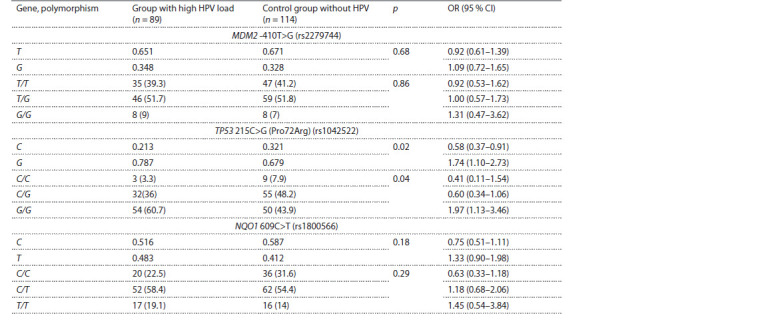
Genotypes (abs., %) and alleles frequencies for MDM2 -410T>G, TP53 215C>G (Pro72Arg), and NQO1 609C>T genes among women with high HPV load and without HPV

An analysis of intergenic interactions showed that the threelocus model of gene interaction has a prediction accuracy of 64 % and cross-validation consistency (10/10) (Table 2). Interaction of TP53 (rs1042522) – MDM2 (rs2279744) – NQO1 (rs1800566) genes is associated with the risk of high HPV load among women (OR = 3.05, 95 % CI = 1.73–5.46; p = 0. 0001).

**Table 2.**
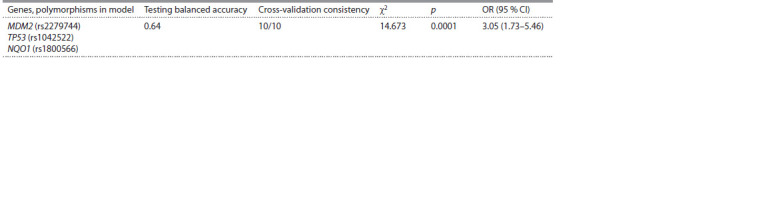
Analysis of intergenic interactions by the multifactor dimensional reduction algorithm (MDR)

A radial diagram demonstrates the contribution of polymorphism of each gene, both individually and in combination with others for the three-loci. In the vertices of the diagram, the values of information for individual genes are indicated, on the edges – the information value of the interaction of a pair of genes. The studied SNPs affect the formation of the viral load to varying degrees. According to the model of loci interaction (see the Figure), the highest predictive potential is possessed by the SPN of the TP53 gene (2.26 %). The TP53 and MDM2 loci have the greatest effect by intergenic interaction. A pronounced synergism was revealed between these loci – the total effect of the combination is 2.87 %. Its information value is higher than the sum of its individual effects.

**Fig. 1.**
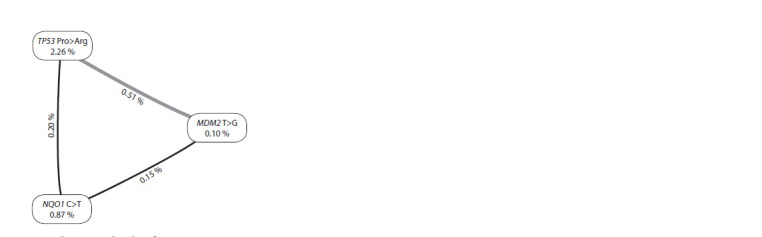
Interaction analysis among three loci of MDM2 -410T>G, TP53 215C>G (Pro72Arg), and NQO1 609C>T of women with high HPV viral load. The informational value of each marker is presented on vertices; the informational value of the interaction for a pair of loci is presented on the edges. The nature of the interaction between genes is shown by the color of the line (gray – pronounced synergism, black – moderate synergism).

## Discussion

Cervical cancer is the most common gynecological cancer among women and the high-risk HPV genotypes play a major role in abnormal lesion development and cervical malignant neoplasms (Malagón et al., 2019; So et al., 2019). The presence of a high viral load in HPV-positive women indicates that the virus has not been entirely removed and will likely continue to replicate in the body cells for a long time. Long-term virus persistence contributes to the incorporation of HPV DNA into the human genome, the expression of E6/E7 oncogenic proteins, and the development of cancer (McBride, Warburton, 2017; Gheit, 2019).

Human papillomatosis appears to be a polygenic disease, suggesting that recurrent, small-effect genetic variations can have consequences for disease susceptibility (Khoury et al., 2018). Tumor development is largely attributed to genetic variations in the host’s cell cycle control (Litwin et al., 2017). The relationships between the TP53 gene (rs1042522), MDM2 gene (rs2279744), NQO1 gene (rs1800566), and the risk of high HPV load have been investigated in this study

In our study, 43.8 % of women (89 out of 203) were positive for high-risk HPV types. Our analysis revealed an association of high viral load formation risk with 215G (72Arg) allele carriage (OR = 1.74, 95 % CI = 1.10–2.73; p = 0.02) and 215GG (72Arg/Arg) genotype of TP53 gene (OR = 1.97, 95 % CI = 1.13–3.46; p = 0.04). On the contrary, 215C (Pro72) allele (OR = 0.58, 95 % CI = 0.37–0.91; p = 0.02) and 215CC (72ProPro) genotype (OR = 0.41, 95 % CI = 0.11–1.54; p = 0.002) showed protective effect compared to the control group. The polymorphic variants of the p53 protein with Pro or Arg in codon 72 have been shown to vary in the efficiency of interaction with the E6 oncoprotein of HPV (Storey et al., 1998). The Arg variant is degraded by the E6 oncoprotein more readily than the Pro variant. Therefore, the carriers of the Arg/Arg genotype have p53 protein more vulnerable to viral protein-induced degradation (Hu et al., 2010). Our results are consistent with several other studies suggesting that HPVpositive women are more vulnerable to cervical malignant neoplasms when having ТР53 72Arg/Arg genotype and ТР53 72Arg allele (Basyar et al., 2016; Moschonas et al., 2017).

MDM2 promotes cell cycle progression through the activation of S-phase, via interaction with the retinoblastoma tumor suppressor protein and the transcriptional factor E2F (Oliner et al., 2016). MDM2 is one of the central nodes in the p53 pathway regulation. It has been shown that even a small change in MDM2 level may affect the p53 pathway and, subsequently, cancer development (Mendoza et al., 2014). Our analysis showed no statistically significant difference in the genotypes ( p = 0.86) and allele frequencies ( p = 0.68) distribution of MDM2 -410T>G gene polymorphism in two women groups.

Our analysis showed no statistically significant difference in the genotypes ( p = 0.29) and allele ( p = 0.18) distribution of NQO1 609C>T gene polymorphism in the two groups of women. In agreement with our results, J. Chansaenroj and his coworkers showed no association of the NQO1 609C>T polymorphism with the risk of cervical cancer (Chansaenroj et al., 2013). At the same time, several studies reported a relationship between NQO1 609TT genotypes and the risk of cervical cancer (Niwa et al., 2005; Yang et al., 2020). The NQO1 gene (rs1800566) TT genotype is associated with null enzyme activity and could influence cancer progression by reducing cytotoxic agents containing the quinone moiety (Diao et al., 2017).

Favorable conditions for HPV persistence include multiple genetic substitutions which result in gene expression changes. In our work, the analysis of gene-gene interactions (MDR) showed significant interaction of the polymorphic loci (OR = 3.05, 95 % CI = 1.73–5.46; p = 0. 0001) for increased viral load (see Table 2). The interaction of the polymorphic variants for the three loci of the genes TP53 215C>G (Pro- 72Arg), MDM2 -410T>G, and NQO1 609C>T are associated with HPV viral load increase.

A synergistic effect was revealed between the studied loci. That is, the combined effect of these loci is more pronounced than individual effects. Thus, we revealed an increased risk of a high viral load in HPV infection in the case of a combination of polymorphic variants of the TP53, MDM2, and NQO1 genes. The risk may be due to disturbances in the work of the checkpoints of the cell cycle due to the activation of the processes of degradation of the p53 protein.

The current study has several limitations. First, the small sample size: our results should be verified in larger populations as well as in other ethnic groups. Second, women with cervical cancer were not included in our research. Comparison of the different histological types of cervical cancer may also be warranted for future studies to determine whether the frequency of TP53, MDM2, and NQO1 gene polymorphisms differ based on the histological types of cervical cancer. Third, the influence of epidemiologic risk factors such as smoking, alcohol intake, and sexual behavior or pathogenic factors like bacteria with the risk of HPV infection was not included. It would be interesting to analyze if TP53, MDM2, and NQO1 production is associated with environmental or pathogenic factors.

## Conclusion

Our results demonstrate that the risk of high viral load formation is associated with TP53 215G (72Arg) allele and TP53 215GG (72ArgArg) genotype in HPV-positive women. Although the individual SNPs of MDM2 -410T>G and NQO1 609C>T genes did not reveal a statistically significant frequency difference in our study, intergenic interactions analysis revealed significant interaction for all polymorphic variants. This demonstrated that the infection development depends on the synergistic effect of several polymorphisms that induce changes in gene expression and represent an allelic load for HPV-positive cells. However, the role of the genetic susceptibility to HPV infections and high HPV load with TP53 rs1042522, MDM2 rs2279744, NQO1 rs1800566 polymorphisms requires further investigation

## Conflict of interest

The authors declare no conflict of interest.
